# Genotoxic and Cytotoxic Effects on the Immune Cells of the Freshwater Bivalve *Dreissena polymorpha* Exposed to the Environmental Neurotoxin BMAA

**DOI:** 10.3390/toxins10030106

**Published:** 2018-03-01

**Authors:** Alexandra Lepoutre, Nadia Milliote, Marc Bonnard, Mélissa Palos-Ladeiro, Damien Rioult, Isabelle Bonnard, Fanny Bastien, Elisabeth Faassen, Alain Geffard, Emilie Lance

**Affiliations:** 1 Université de Reims Champagne-Ardenne UMR-I 02 INERIS-URCA-ULH SEBIO Unité Stress Environnementaux et, BIOsurveillance des milieux aquatiques UFR Sciences Exactes et Naturelles, Campus du Moulin de la Housse, BP 1039 51687 Reims CEDEX, France; alexandra.lepoutre@univ-reims.fr (A.L.); nmilliot@edu.mnhn.fr (N.M.); marc.bonnard@univ-reims.fr (M.B.); melissa.palos@univ-reims.fr (M.P.-L.); damien.rioult@univ-reims.fr (D.R.); isabelle.bonnard@univ-reims.fr (I.B.); fanny.donaz@univ-reims.fr (F.B.); alain.geffard@univ-reims.fr (A.G.); 2 RIKILT, Wageningen Research, Akkermaalsbos 2, 6708 WB Wageningen, The Netherlands; els.faassen@wur.nl; 3 Aquatic Ecology and Water Quality Management Group, Wageningen University, Droevendaalsesteeg 3a, 6708 PB Wageningen, The Netherlands

**Keywords:** BMAA, hemocytes, genotoxicity, cytotoxicity, immunotoxicity, freshwater bivalves

## Abstract

The environmental neurotoxin β-*N*-Methylamino-l-alanine (BMAA) has been pointed out to be involved in human neurodegenerative diseases. This molecule is known to be bioaccumulated by bivalves. However, little data about its toxic effects on freshwater mussels is available, particularly on the hemolymphatic compartment and its hemocyte cells involved in various physiological processes such as immune defenses, digestion and excretion, tissue repair, and shell production. Here we exposed *Dreissena polymorpha* to dissolved BMAA, at the environmental concentration of 7.5 µg of /mussel/3 days, during 21 days followed by 14 days of depuration in clear water, with the objective of assessing the BMAA presence in the hemolymphatic compartment, as well as the impact of the hemocyte cells in terms of potential cytotoxicity, immunotoxicity, and genotoxiciy. Data showed that hemocytes were in contact with BMAA. The presence of BMAA in hemolymph did not induce significant effect on hemocytes phagocytosis activity. However, significant DNA damage on hemocytes occurred during the first week (days 3 and 8) of BMAA exposure, followed by an increase of hemocyte mortality after 2 weeks of exposure. Those effects might be an indirect consequence of the BMAA-induced oxidative stress in cells. However, DNA strand breaks and mortality did not persist during the entire exposure, despite the BMAA persistence in the hemolymph, suggesting potential induction of some DNA-repair mechanisms.

## 1. Introduction

Harmful algal blooms (HAB) are increased by human activities [1] and, because of their ability to produce secondary metabolites that can be harmful to other species, they are known to have an impact on ecosystems and human health [2]. Among those toxins, the neurotoxin β-*N*-Methylamino-l-alanine (BMAA), a hydrophilic non-protein amino acid, is a subject of growing concern. Indeed, BMAA could be potentially involved in the development of human neuro-degenerative pathologies such as the Amyotrophic Lateral Sclerosis-Parkinsonism-Dementia complex (ALS-PDC) [3]. Historically, the etiological link between BMAA and ALS has been pointed out in a study conducted in Guam in the 50 s, where the incidence of ALS was 50 to 100 times higher than in the rest of the world [3]. As BMAA has been reported in human brains of people who suffer from ALS-PDC [4], it has been alleged that the contamination came from chronic exposure through a biomagnification process. Indeed, the endosymbiotic cyanobacteria of the genera *Nostoc*, present in cycads roots, has been shown to produce BMAA [3,5]. The cycads seeds are then ingested by fruits bats *Pteropus mariannus* while foraging. As bats were traditional food items on Guam, human contamination is thought to have occurred during the consumption of whole individuals [3,5]. However, this hypothesis is subject to controversy [6]. The BMAA toxic mechanism has not been fully determined, but current research shows that the neurotoxin may (i) form carbamate adducts when conjugated with bicarbonate ions at physiological concentrations, inducing a motoneuron excitotoxicity via the L-glutamate receptors [7,8,9], which can lead to an oxidative stress an cell death [8,9]; (ii) inhibit the synthesis and/or the stimulation of glutamine degradation [10]; (iii) inhibit cells cysteine/glutamate antiporter, leading to a depletion of glutathione (GSH) which increases oxidative stress [8]; and (iv) bind to or incorporate into proteins during their synthesis [11], although this hypothesis has been criticized [12]. Little data about animal toxicology or *in situ* occurrence are available for this emerging environmental neurotoxin, which is associated with controversies in relation to analytical challenges for its detection and quantification in various matrices [13].

BMAA was historically thought to be produced ubiquitously by cyanobacteria; recent upgrading of analytical methods has narrowed the list of cyanobacterial BMAA producers and shown that it is also produced by some diatoms and dinoflagellate species [14,15,16,17,18]. The neurotoxin has also been detected in food webs outside of Guam, in animals destined for human consumption (fish, mussel, and oyster) in China, United States, and Europe [15,16,19,20,21,22,23,24]. Moreover, a possible cluster of ALS has been pointed out in the Thau Lagoon (France) that could be potentially linked with the consumption of BMAA-contaminated seafood [25].

Up to now, BMAA bioaccumulation following laboratory exposure of invertebrates to dissolved BMAA or to BMAA-producers has been demonstrated in gammarids [26,27,28] and bivalves [29,30], including the freshwater mussel *Dreissena polymorpha* [29] investigated in the present study. The freshwater sessile bivalve *D. polymorpha*, is native from the Ponto-Caspian seas and is now a widespread species [31]. Bivalves are commonly used as bioindicators and biomonitors in relation to their direct contact with medium, their abundance, broad distribution, limited mobility, lifespan, and ease of handling [32]. This mussel can filter particles from 0.4 µm [33] to 200 µm [34] or even 750 µm after 16 h of starvation [35], including phytoplankton species, and is also known to uptake efficiently amino acid from its environment [36]. This wide food size range combined with filtering capacities ranging from 5 to 400 mL/mussel/h [37] allows zebra mussels to be potentially intoxicated by BMAA through three main pathways: (i) the gill absorption of dissolved BMAA; (ii) the ingestion of food particles in which BMAA may be adsorbed; and (iii) the ingestion of BMAA-producers. Those contamination pathways, and more particularly the gill diffusion, may induce a BMAA distribution in the hemolymphatic compartment.

Bivalves have an open circulatory system composed of a heart and vessels in which the hemolymph circulates. Hemolymph is compossed of cells named hemocytes and dissolved proteins. Those cells are primarily in charge of the immune defense and pathogens elimination [38], but also take part in various physiological processes including digestion, tissue repair, shell production, and excretion [38,39,40]. Because of their involvement in mussels defense and homeostasy, hemocytes have a key role in bivalves physiology. The aim of this study was to assess the impact of the BMAA on hemocytes of *D. polymorpha*, in terms of cell mortality, phagocytosis, and DNA integrity, through a 3-week discontinuous exposure of *D. polymorpha* to dissolved BMAA, followed by 2 weeks of depuration. Due to the lack of a known organism that could produce BMAA steadily, zebra mussels were exposed to dissolved BMAA to a concentration 7.5 µg of dissolved BMAA/mussel/3 days. Few data of environmental BMAA concentrations acquired with a reliable analytical method and reported per liter of water are available to date [41]. One study reports BMAA concentrations from 0.01 to 0.3 µg/L in lake samples (Canada) [42]. Based on the filtering capacity of *D. polymorpha*, ranging from 0.12 to 9.6 L/mussel/day according to food concentrations [43], one zebra mussel could theoretically filtrate from 1.2 ng BMAA/day to 9.6 µg BMAA/day. This broad range of BMAA environmental concentrations in water includes the one used in this study. As the BMAA pathway into mussels is still unknown, the hemolymphatic concentration of BMAA was followed throughout the study to assess hemocytes exposure. We discuss the BMAA presence in the hemolymph and its impacts on hemocytes, in terms of potent cell penetration and alteration of DNA integrity, as well as subsequent potent DNA reparation. 

## 2. Results

In this study, freshwater mussels *D.polymorpha* have been exposed to 7.5 µg BMAA/3 days for 21 days, followed by 21 days of depuration in clear water.

### 2.1. BMAA Accumulation in Hemolymph

**Figure 1:**
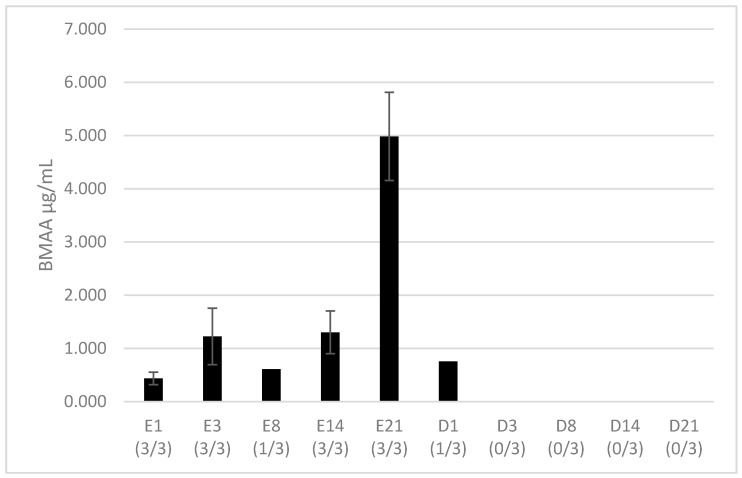
Concentration of BMAA (µg/mL) in hemolymph samples during the exposure (E) and depuration (D) periods; numbers in brackets represent the number of samples in which BMAA quantification has been performed compared to the three samples analyzed. Technical difficulties prevented BMAA quantification in the other samples, although BMAA was detected. No significant difference between samples appeared with a Mann-Whitney test, *p* < 0.01.

The total BMAA fraction was evaluated here, comprising the free BMAA, soluble in sample supernatant, and the BMAA associated/bound to proteins or peptides of low molecular weight that requires tissue hydrolysis to be extracted [44]. Total BMAA was detected in all hemolymph samples. However, a strong suppression of the internal standard (D_3_BMAA) signal was observed in some samples (2 samples from day 8 of exposure, 2 from day 1 of depuration, and all other depuration samples), which made it impossible to accurately estimate the BMAA concentration in these samples. Therefore, only the data that are quantified based on the internal standard response are shown. After 24 h of exposure, total BMAA has been found in all hemolymph samples with a mean of 0.438 ± 0.117 µg/mL (Figure 1). Then, the concentration in samples remained constant until day 21 of exposure, in which the concentration increased (*p* > 0.01) and reached 4.984 ± 0.829 µg/mL. The day after, representing the first day of depuration, BMAA could only be quantified in one sample among three at a concentration of 0.755 µg/mL.

### 2.2. Hemocytes Mortality

**Figure 2:**
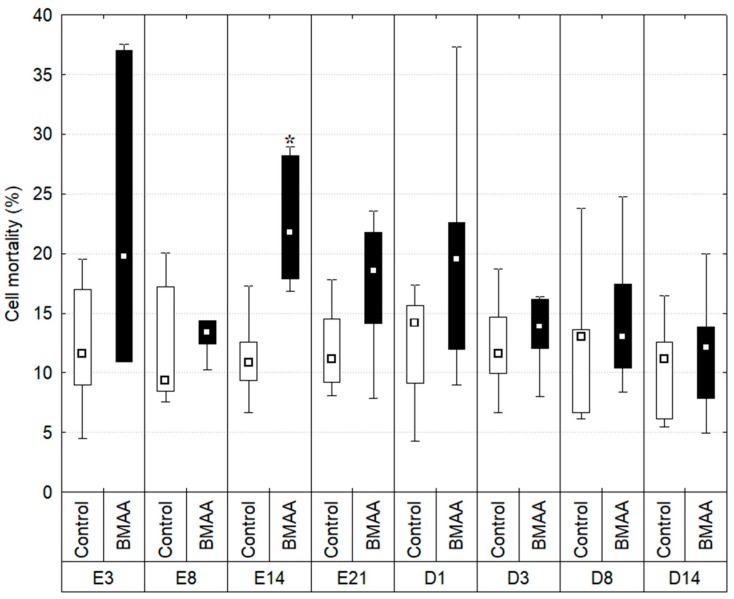
Hemocyte mortality (%) measured during the exposure (E) and depuration (D) period. Boxplots indicate first and third quartile of the observations, whiskers indicate minimum and maximum values, and the median is indicated by a square. Dark bars indicate the BMAA treatment (7.5 µg of dissolved BMAA/mussel/3 days) and the white bars the controls. * Indicates a significant difference between the exposed individual and the control of the same sampling date with a Mann-Whitney test, *p* < 0.01, n between 6 and 10. Due to a technical issue, data from E1 are not shown.

The percentage of dead hemocytes has been measured throughout time (Figure 2).

The percentage of mortality in controls remained stable during the experiment: a minimum of 11.513% ± 1.118 of dead cells observed on day 14 of the exposure period, and a maximum of 12.702% ± 1.806 on day 8 of the depuration period (Kruskal-Wallis test: *p* > 0.01). The percentage of mortality from individuals exposed to BMAA did vary during the experiment. On day 3 and 8 of exposure the mortality remained stable (22.672% ± 12.719 on day 3 and 13.540% ± 2.600 on day 8) and did not vary significantly from controls (*p* > 0.01; *U* values respectively 11 and 9). Then, the percentage of dead hemocytes on day 14 of exposure is significantly higher than the one observed in controls (*p* = 0.002, *U* = 1) and reached 22.582% ± 5.464. Data also shows greater inter-individual variations in individuals exposed to BMAA compared to controls at all sampling times, except at day 8 of exposure.

### 2.3. Hemocytes Phagocytosis

**Figure 3:**
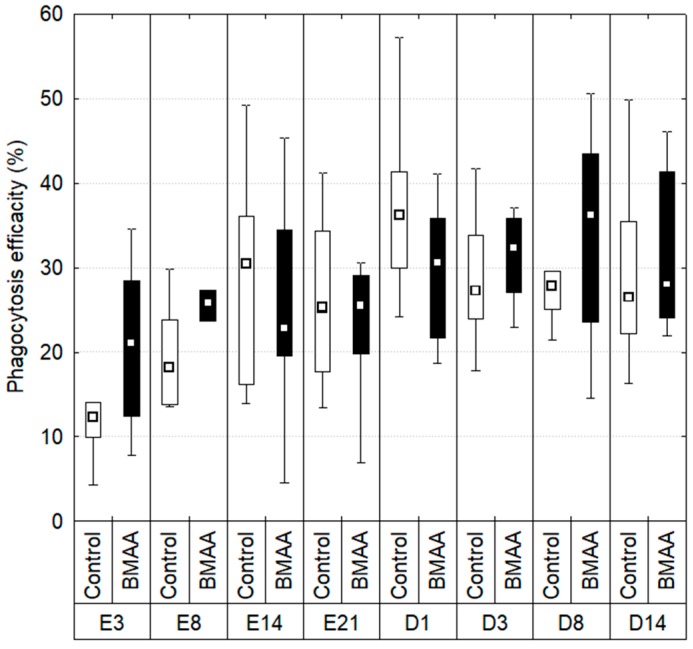
Phagocytosis efficiency measured during the exposure (E) and depuration (D) periods. Boxplots indicate first and third quartile of the observations, whiskers indicate minimum and maximum values, and the median is indicated by a square. Dark bars indicate the BMAA treatment (7.5 µg of dissolved BMAA/mussel/3 days) and the white bars the controls. No difference between exposed individual and controls of the same time with a Mann-Whitney test, *p* < 0.01, *n* between 6 and 10.

During the exposure, the phagocytosis efficiency was evaluated through the measure of the percentage of hemocytes that had engulfed 3 beads ore more (Figure 3). The data did not show significant differences or trends between controls and mussels exposed to BMAA during the exposure and the depuration periods (Kruskal-Wallis test: *p* < 0.01).

**Figure 4:**
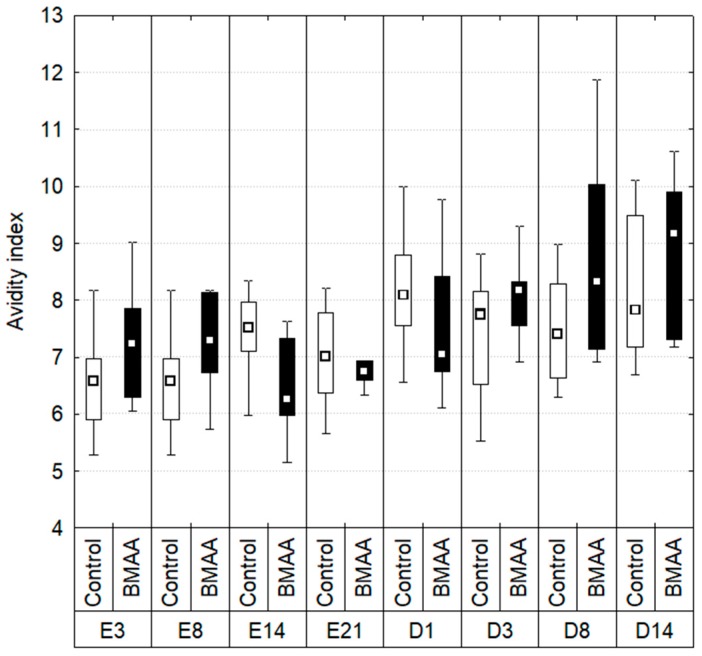
Avidity index measured during the experiment. (%) measured during the exposure (E) and depuration (D) periods. Boxplots indicate first and third quartile of the observations, whiskers indicate minimum and maximum values, and the median is indicated by a square. Dark bars indicate the BMAA treatment (7.5 µg of dissolved BMAA/mussel/3 days) and the white bars the controls. No difference between exposed individual and controls of the same time with a Mann-Whitney test, *p* < 0.01, *n* between 6 and 10.

The mean number of beads engulfed per cell, representing the avidity index (Figure 4), was also measured. As for the phagocytosis efficiency, no significant differences appeared between controls and individuals exposed to BMAA during the exposure and depuration periods (Kruskal-Wallis test: *p* > 0.01).

### 2.4. DNA Integrity 

**Figure 5:**
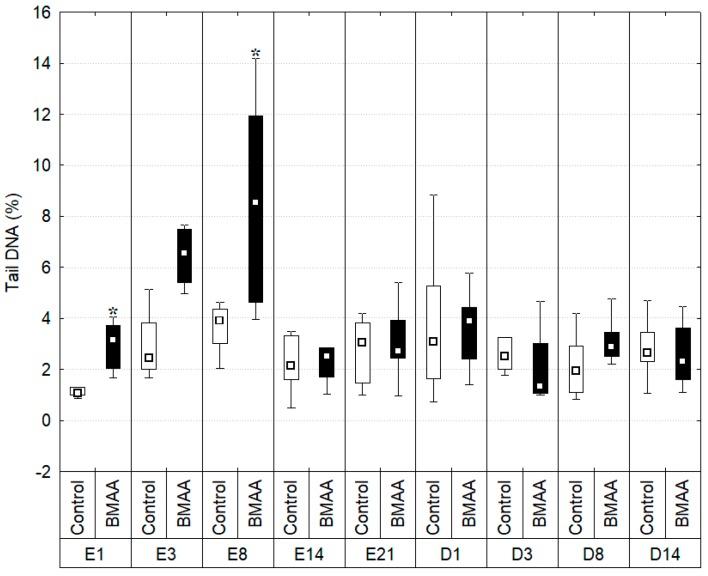
Mean tail DNA (%) measured during the exposure (E) and depuration (D) periods. Boxplots indicate first and third quartile of the observations, whiskers indicate minimum and maximum values, and the median is indicated by a square. Dark bars indicate the BMAA treatment (7.5 µg of dissolved BMAA/mussel/3 days) and the white bars the controls. * Indicates a significant difference between the exposed individuals and the controls of the same sampling date with a Mann-Whitney test, *p* < 0.01, *n* = 8.

The percentage of DNA present in the tail of comets (Tail DNA), reflecting the degradation of DNA, has been studied throughout this experiment (Figure 5). 

DNA strand breaks in controls remained stable over the experiment (Kruskal-Wallis test: *p* > 0.01) with a minimum of 1.212 ± 0.379% of tail DNA at the first day of exposure and a maximum of 4.018% ± 4.369 after 21 days of exposure. On the other hand, greater variations have been observed in individuals exposed to BMAA. At the first day of exposure, the percentage of tail DNA (% tDNA) in individuals exposed to BMAA reached 2.942% ± 0.927 and was significantly higher than the one of controls (*p* = 0.003, *U* = 1). However, it should be notice than the % tDNA in controls tended to be lower on the first day of the experiment compared to other sampling dates, although the difference was not significant (*p* > 0.01). This low control value on day 1 revealed significant DNA damage in BMAA-exposed individuals, while their % tDNA values were comparable to those of controls at following times. Therefore, we do not put emphasis about the significant DNA damages observed at this first day of exposure. Then, DNA integrity of exposed individuals decreased throughout time, reaching a maximum of DNA strand breaks at the eighth day of exposure, with significantly higher damages, 9.748% ± 6.617, than in controls (*p* = 0.0087, *U*= 7). It should be noticed that, even if not significant (*p* = 0.012, *U* = 8), a trend of DNA damages in exposed organism has been observed at the third day of exposure in a higher level (7.290% ± 3.034) compared to controls (3.801% ± 3.554).

From day 14 of exposure, the level of DNA strand breaks in hemocytes of exposed mussels remained low and similar to controls during the rest of the exposure period, as well as during the depuration period (Kruskal-Wallis test: *p* > 0.01).

## 3. Discussion

Through a 3-week discontinuous exposure of the freshwater bivalve *D. polymorpha* to 7.5 µg BMAA/individual/3 days, the objectives of the experiment consisted of analyzing the presence of BMAA (total fraction) in the hemolymphatic compartment and studying potent subsequent impact on hemocytes in terms of (i) cytotoxicity; (ii) immunotoxiciy; and (iii) genotoxicity. As no steady laboratory BMAA-producer has been identified to date, zebra mussels were exposed to dissolved purified BMAA.

The potent immunotoxicity of BMAA has never been studied so far. It is interesting here to notice that BMAA was detected in the hemolymph from the first day of exposure until the last day of depuration, confirming the BMAA penetration in this compartment during in-vivo exposure. Another point is the chronology of the impact of BMAA on the hemocytes of *D. polymorpha*. Indeed, on the third and eight days of exposure, BMAA induced significant DNA damages, while cell mortality remained similar to controls, suggesting the genotoxicity of this molecule on hemocytes. Then, on the fourteenth day of exposure, significant hemocyte mortality was observed in BMAA-exposed cells, compared to controls, while no difference in the BMAA content in hemolymph was noticed compared to previous times of exposure, and no significant individual mortality was observed. This observed hemocyte mortality could potentially be a result of previous DNA damages. At the last day of exposure, BMAA concentration in hemolymph seemed to increase compared to previous days, but no genotoxicity or cytotoxicity were observed anymore.

Mussels have already been shown to accumulate BMAA during laboratory exposures to dissolved BMAA, but the transfer of BMAA from the medium to the hemolymphatic compartment remains unknown. To date, only the presence of BMAA in the entire mussel body has been demonstrated during an exposure of the marine mussel *Mytilus galloprovincialis* to dissolved BMAA (1, 2.5 and 5 mg/L) for 24 and 48 h and to two BMAA-producing cyanobacterial strains (*Synechocysti salina* and *Microcoleus chthonoplastes*) [30]. Moreover, an exposure of *D. polymorpha* to approximately 100 µg/L of isotopically labeled BMAA for 48 h followed by 24 h of depuration has shown that BMAA was taken up by zebra mussels, and that nearly all of the exogenous-labelled BMAA available has been removed from the medium [29]. In the present study, BMAA dissolved in the medium was later quantified in the hemolymphatic compartment, suggesting two entrance pathways: (i) a gill diffusion directly from the medium to the hemolymph; or (ii) an ingestion through a BMAA adsorption on food particles (*Chlorella* sp. and *Scenedesmus* sp.). However, because mussels feeding and BMAA addition in the medium has been done at different days, this hypothesis seems unlikely. Under such hypotheses, hemocytes may have been in contact with BMAA during the digestion and/or nutrient transport, through the digestive system. However, no information is available concerning either BMAA bioaccessibility (i.e., the BMAA fraction extracted from food after digestion that is available for absorption by the hemolymphatic compartment) or its biodisponibility (i.e., the BMAA fraction that indeed reaches the hemolymphatic compartment). This study, however, confirmed the BMAA transfer from the medium to the hemolymph of mussels. The hemolymph sampled in the posterior adductor muscle represents one fraction of the whole hemolymphatic compartment (the rest being in contact with organs), and the data provided from this analysis only allows the assessment of the presence of total BMAA in this tissue and the subsequent exposure of immune cells contained in it. As the BMAA content in hemolymph remained stable and did not increase in a time-dependent manner from the first day of exposure to day 14, we may hypothesize that hemolymph only transports BMAA into mussel’s organs and therefore does not accumulate this toxin, which remains to be investigated. The increase of the total BMAA content (free or bound to peptides or to proteins) in the hemolymph on day 21 of exposure also required further investigations. The total BMAA content measured on day 21 might have been metabolized and emitted by some other organs as a degradation product or conjugated molecule for elimination. DNA damages and hemocytes mortality observed in this study suggest that the hydrophilic molecule BMAA may cross the cytoplasmic membrane. As amino-acids diffusion into cells is a minor pathway at physiological concentrations [45], it could be hypothesized that some transporters may allow this toxin to cross membranes. So far, as described in vertebrates’ nervous system, pathways that could potentially explain BMAA entrance into cells are (i) the cerebrovascular large neutral amino acid carrier, which allows BMAA to cross the blood-brain barrier in rats [46]; and (ii) the cystine/glutamate antiporter (system xc-), which allows BMAA to cross cells in rats cortical cells cultures [47]. Hemocytes membranes might therefore also have amino-acid transporters that are able to get BMAA into the cell. So far, except for one study carried out on bacteria [48], toxicological studies about the impact of BMAA have been carried out on vertebrates, and the extrapolation of potential mechanisms of action on invertebrates remains tenuous. As BMAA is an emerging environmental neurotoxin, little evidence of animal toxicology is available to date, and its metabolites are also unknown; therefore, it may be difficult to assess if the observed damages where caused by this molecule or its metabolites. DNA damages by BMAA could have been induced either indirectly on the genome or directly by crossing the nuclear membrane. An Ames test on five *Salmonella typhimurium* strains with five BMAA concentrations ranging from 11 to 900 µg/plate has shown that BMAA did not induce any mutation and was neither cytotoxic nor genotoxic for bacteria [48]. A study carried out on human neuroblastoma cell line SH-SY5Y [49] showed, through a plasmid assay, that BMAA is able to induce DNA strand breaks from 309.2 mg/L, potentially linked with the nitrosation of BMAA, which generates an alkylating form that is also able to induce more DNA strand breaks than BMAA itself. DNA damages and cell mortality observed here could also be explained by the ability of BMAA to induce an oxidative stress in cells and/or its potential insertion in or binding to proteins. The induction of an oxidative stress by BMAA has been shown in murine cortical cell cultures; in presence of bicarbonate at physiological concentration, BMAA could form a carbamate adduct that is a glutamate analogue [7]. Under this form, this molecule may thus be able to activate glutamate receptors in motoneurons of the central nervous system, provoking a cascade of reaction leading to excitotoxicity, oxidative stress, and cell death [8,9]. Through the inhibition of cystine/glutamate antiporter in cortical cultures, BMAA could also inhibit the cystine uptake leading to a depletion of glutathione (GSH), thus increasing cellular oxidative stress [8]. Glial cells exposed to 0.5–1–3 mM BMAA [50], with and without bicarbonate, showed a relative generation of reactive oxygen species (ROS) compared to untreated samples. At the individual level, the exposure of *D.polymorpha* to 10–100 and 500 µg/L BMAA during a maximum period of 48 h showed no significant alteration in activities of antioxidant enzymes catalase (CAT), glutathione reductase (GR), glutathione peroxidase (GPx), or of the biotransformation glutathione S-transferases (GST) [29]. However, variations of activities of antioxidants enzymes might have been observed in *D. polymorpha* if the study covered a longer period of time. Nevertheless, in the same study, BMAA induced an oxidative stress (i.e., increase of CAT and GR activities) on other freshwater bivalves (*Unio tumidus* and *Corbicula javanicus*) after 24 h and 48 h of exposure, respectively [51]. Oxidative stress has been shown to induce DNA damages that can lead to apoptosis in eukaryotes [52] and could also stress mitochondria, which could lead to DNA damage and even cell death [50]. This oxidative stress might be the first possible pathway of the genotoxicity observed in this study. The mechanism of DNA damages induced by BMAA would deserve to be elucidated by further investigations. In order to study if an oxidative stress is involved in the DNA damages during a BMAA exposure, it could be interesting to use oxidative probes such as DHR-123 [53] or to analyze markers such as 8-hydroxyguanosine, which is used to measure the rate of oxidative damage to nucleic acids and lipids [54]. Also, it could be interesting to use different versions (standard alkaline, modified-) of the comet assay to highlight DNA strand breaks or oxidative DNA lesions.

Another mechanism that could explain the observed DNA damages is the potential insertion of BMAA into proteins. Indeed, a BMAA insertion into proteins would lead to their misfolding and a subsequent change of their properties, directly or indirectly inducing stress. Exposure of human cell lines MCR-5 and SH-SY5Y to 31 nM of ^3^H-BMAA and 200–1000 μM of BMAA showed that BMAA was associated with the protein fraction, and this association was positively correlated with protein synthesis. Moreover, the addition of serine in the medium leads to a decrease in the detection of BMAA in the protein fraction [11]. The authors hypothesized that BMAA was misincorporated in human cells in place of the amino acid serine, a phenomenon that could lead to protein misfolding and therefore to a potential cell dysfunction [11]. However, this hypothesis is still a matter of debate and there is yet no evidence that BMAA could be integrated into proteins rather than chemically associated with them [12]. Moreover, this integration into proteins or adhesion might be limited in time: BMAA was found in neonatal rats of 9 to 10 days, 24 h after 2 days of subcutaneous injection of a dose corresponding from 50 to 600 mg/kg BMAA HCl, but no BMAA was detected at 28 weeks of age [55]. BMAA misincorporation could elicit a stress of the endoplasmic reticulum which, when the accumulation of misfolded protein is excessive, could lead to organelle dysfunction and trigger cell death [56]. If proteins containing proteomimetic amino acids are efficiently degraded or generate truncated proteins, they can give rise to peptides that can induce autoimmune symptoms due to the recognition as non-self-antigens [57].

In this study, the phagocytosis avidity (mean number of beads phagocyted per hemocytes) and efficiency (mean number of hemocytes that have phagocyted three beads and more) did not differ in mussels exposed to BMAA compared to controls. This could suggest that the immune system of bivalves was not activated by BMAA, because it did not recognize this molecule or its metabolites, which is expected for an amino acid. Indeed, hemocytes commonly take charge of particles of the size range of bacteria [58]. On the other hand, it would suggest that the toxin did not interfere with bead phagocytosis by hemocytes, despite the induced genotoxicity on days three and eight of exposure and subsequent cell mortality observed on day 14 of exposure. The absence of impact on both phagocytosis indexes, despite an increase of cell mortality, could be explained by the fact that analyses of phagocytosis have been performed only on cells that were alive. Indeed, those measurements were done with the use of FITC-labelled beads, without the use of a dye that quantifies dead cells, and were performed on events that had the same size and complexity as hemocytes. Because dead cells become smaller and less complex, their signal might be out of our acquisition area. Also, cell mortality has been measured through the use of propidium iodide (PI), a stain excluded by intact membranes. This dye does not rapidly cross intact cells membranes and, if it manages to penetrate them, the molecule is likely to be pumped outside [59]. When membrane integrity is compromised, PI can bind itself non-selectively to nucleic acids [59]. While propidium iodide is used to distinguish living cells from dead ones [60], this dye, in fact, assesses membranes integrity, which reflects cell mortality. Thus, using this probe is only informative of the percentage of cells that are dead. It has been observed that *Saccharomyces cerevisiae* cells can take up PI while undergoing a stress, but membranes of a subpopulation of cells could be later repaired [61]. Thus, it is possible that the increase of PI fluorescence on day 14 of exposure reflects the mortality of most cells but a reversible transient permeability for others, caused by a BMAA-induced stress.

The absence of genotoxic or cytotoxic effects on day 21 of exposure, while zebra mussels are still exposed to BMAA, could potentially be explained by the establishment of mechanisms that allows hemocytes to remain viable, and functioning, while undergoing a contamination, and/or a BMAA detoxification/metabolization. A mechanism that could explain DNA reparation in this context could be the base excision repair (BER) pathway [52,62]. First, the abnormal base is detected and excised by DNA glycosylases. Then, the remaining sugar is cut, and an undamaged nucleotide is inserted, correcting the site. The BER pathway exists in bivalves and is known to act fast. Indeed, it has been shown that an *in vivo* exposure of the freshwater mussels *Limnoperna fortunei* to pentachlorophenol (PCP) leads to DNA damages that were repaired after 2 h in clean water [63]. If measured genotoxicity and cell death are triggered by protein misfolding, cells could potentially activate the unfolded protein response (UPR) pathways. UPR is a collection of signaling pathways that leads at first to a transient attenuation in the rate of protein synthesis, then an upregulation of genes encoding proteins involved in protein folding, and lastly to the degradation of endoplasmic reticulum-localized proteins [56]. 

Moreover, as discussed above, the total BMAA measured in the hemolymph on day 21 could have been metabolized or be a degradation product emitted by other organs, and therefore could have lost its toxicity, explaining the absence of effect. This has to be investigated.

Finally, the high dispersion of boxplots showing results of DNA damages and hemocytes mortality in individuals exposed to BMAA can be explained by inter-individual differences in the ability to (i) uptake BMAA from the medium. Indeed, valves movement of mussels is asynchronous and filtration rate may considerably vary between individuals (0.1 to 0.4 L/mussel/h) [43]; (ii) accumulate, metabolize, and eventually detoxify BMAA; and (iii) cope with potent oxidative stress and DNA damages induced by the neurotoxin. 

## 4. Conclusions

Through an in vivo exposure of a freshwater mussel, this study constitutes the first evidence that BMAA is present in the hemolympatic compartment of mussels, and induces transient DNA damages on hemocytes, which may lead to a short time cellular death. However, whether this hydrophilic amino acid has directly induced DNA strand breaks and cell cytotoxicity, or indirectly via pathways such as oxidative stress or protein misfolding, remained to be investigated. Also, the kinetics of BMAA accumulation and elimination in zebra mussels remains to be deeply investigated via a follow-up of the BMAA content in several organs.

## 5. Materials and Methods

### 5.1. Mussels Sampling and Acclimation

Zebra mussels were collected at the same spot, at about 5 m depth in March at the Lac-du-Der-Chantecoq (48°36′07.7″ N; 4°44′37.0″ E). In the laboratory, mussels were kept 1 day at 4 °C and in the dark in tanks aerated by an air pump (Zolux, Clisson, France) containing 50% of lake water and 50% of Cristalline^®^ (Saint Yorre, France) source water. Mussels measuring 2 ± 0.3 cm were kept acclimating in the dark only in aerated Cristalline^®^ water at 16 ± 2 °C. They were fed with 2 × 10^6^ cells/individual/day of a 50:50 mix of *Chlorella vulgaris* and *Scenedesmus obliquus* (Greensea, Mèze, France). Algal density was measured with an optic microscope (Primovert, Zeiss, Oberkochen, Germany) and KOVA^®^ slides (Kova slide, VWR, Fontenay-sous-Bois, France)

During the 2 week long acclimation, mussels were randomly divided in two groups in duplicate tanks of 275 individuals that contained 3 L of water and aerated with an air pump. Tanks were put at 16 ± 2 °C with a cycle of 12 h of light and 12 h of darkness. The water was changed every 3 days in order to have the same rhythm as during the exposure.

### 5.2. Exposure

Mussels were exposed for 21 days to dissolved BMAA (L-BMAA hydrochloride B-107, Sigma–Aldrich^®^, Saint-Louis, MO, USA) at a concentration equivalent to 7.5 µg BMAA/mussel/3 days in two tanks aerated by an air pump (Zolux, Clisson, France) and containing 275 individuals at the beginning of the experiment. Two 3 L control tanks that contained the same number of mussels put in clear water were added. The water was renewed at 3 days of interval in control and exposure treatments, and mussels were fed similarly to during the acclimation period (with 50:50 mix of *Chlorella vulgaris* and *Scenedesmus obliquus*). BMAA spiking was performed at each water change, at 3 days of interval, but mussels were fed two days after it (i.e., one day before water changing) in order to avoid adsorption of BMAA to the algal food. Mussels were randomly collected and sacrificed at 0, 1, 3, 8, 14, and 21 days of the exposure period and at 1, 3, 8, 14, and 21 days of the depuration period to perform flow cytometry tests (*n* = 10), COMET assay (*n* = 8) and measurement of the BMAA content in hemolymph (*n* = 3 pools of 2 individuals). During the exposure period, the total amount of BMAA spiked at each water change was adjusted for the number of remaining individuals in order to maintain the exposure concentration of 7.5 µg BMAA/mussel/3 days.

### 5.3. BMAA in Hemolymph

Mussels used for the determination of the BMAA content in hemolymph were placed 24 h in clear water before sampling. This has been done to ensure an evaluation of the BMAA fraction remaining in the hemolymph and not the fraction that would have been early metabolized by individuals or possibly released into the environment. The hemolymph was withdrawn from the posterior adductor muscle using 300 µL-Myjector^®^ (Terumo, Tokyo, Japan) syringes, transferred into Eppendorf^®^ tubes (Eppendorf, Hamburg, Germany), and then put in liquid nitrogen and stored at −80 °C. After being freeze-dried, tissues of 2 individuals were pooled. Total BMAA extraction was done according to previous studies [44]. Briefly, after the addition of D_3_BMAA as an internal standard, samples were dried under vacuum then hydrolyzed for 20 h at 105 °C after the addition of 6 M HCl. After a drying step, samples were resuspended in 67% ACN/33% water/0.1% formic acid and analyzed by UHPLC-MS/MS. UHPLC-MS/MS analysis was performed as described in [64] on the same type of equipment but using a 150 mm column instead of a 100 mm column with a 5 mm precolumn. BMAA was well separated from DAB and AEG; the retention time of BMAA and D3BMAA was 9.4 min, the retention time of DAB was 10.8 min, and the retention time of AEG was 11.8 min. For BMAA, the ion ratios were as follows: *m*/*z* 102:88, 27%; *m*/*z* 102:76, 27%; *m*/*z* 102:73, 20%; and *m*/*z* 102:44, 52%. For D3BMAA, the ratios were *m*/*z* 105:88, 31%; and *m*/*z* 105:76, 23%. A 20% relative deviation from these ratios was allowed in the samples. Chromatograms of an analytical standards mixture (Figure S1) and a hemolymph sample (Figure S2) are provided as supplementary information.

Retention times and ion ratios were comparable to those reported in [64]. BMAA concentrations were corrected for the signal intensity of the internal standard. The concentration of BMAA in the hemolymph (in µg/µL fresh tissue) was calculated from the concentration in freeze-dried tissue using a previously established relation between hemolymph fresh weight and weight after freeze-drying. As gradation indicated by syringe is not considered precise enough for an exact quantification of the volume sampled, the weight of the hemolymph sample was evaluated. For this, an evaluation of the FW/DW ratio of the hemolymph was performed beforehand. This was done while taking hemolymph from 5 individuals and weighing it. The FW/DW ratio is 275.63 ± 21.93.

### 5.4. Preparation of Samples for Flow Cytometry and COMET Assay Analysis

Hemolymph was withdrawn as described previously, but syringes were coated with L15-15% medium. The medium was prepared as described in [65].

Samples were kept individually in DNase RNase Eppendorf^®^ tubes that were resting on ice to prevent the formation of aggregates. Cell numeration was performed with an optic microscope (Primovert, Zeiss, Oberkochen, Germany) and KOVA^®^ slides (Kova slide, VWR, Fontenay-sous-Bois, France) using 10 µL of hemolymph. During this step, samples were sorted to discard samples that contained other cellular types than hemocytes. Then, each hemolymph sample was divided in two in order to perform the COMET assay and the flow cytometry analysis on the same individual.

#### 5.4.1. Flow Cytometry

Analyses were performed with a BD Accuri^®^ C6 (Biosciences, Le Pont de Claix, France). A volume corresponding to 5 × 10^4^ hemocytes was used to study the phagocytosis, and an aliquot of the same volume was used to study the mortality. Hemolymph samples were put individually into a 96 well plate. To each sample was added L15-15% medium. Then, the plate was incubated 4 h at 16 °C. 15 min before the end of the incubation, cells were detached with a 1 × trypsin/EDTA mix and incubated 1 min in the dark at room temperature.

- Mortality assay

To study the cell mortality, 1% of propidium iodide (IP, Sigma-Aldrich^®^, Saint-Louis, MO, USA) was added. Propidium iodide is excluded by live cells but enters permeabilized cells and binds DNA by intercalating between the bases; it produces fluorescence that was measured at 670 nm.

- Phagocytosis efficacity and avidity index

Cells samples were put individually into a 96 well plate. To each sample was added L15-15% medium, and FITC-labelled beads of 2 µm diameter were added to have a ratio of 50 beads per hemocyte. The plate was then incubated for 4 h in the dark at 16 °C. Fifteen minutes before the end of the incubation, cells were detached with a trypsin solution as described before. The fluorescence was measured at 530 nm. The phagocytosis efficacity is represented by the percentage of cells that have phagocytosed 3 beads or more; the avidity index is represented by the number of beads phagocyted on average per individual.

#### 5.4.2. DNA Integrity Measurements

Due to technical constraint, the measure of DNA strand breaks has been performed on 8 of the 10 sampled individuals. Thus, those containing gametes have been set aside.

DNA strand breaks were quantified by the alkaline version (pH > 13) of the comet assay, as described [66] with minor modifications. All steps described below were performed under an inactinic light and at 4 °C to prevent any additional DNA damage. The experimental controlconsisted of a THP-1 cell line, provided by ATCC^®^ (Manassas, VA, USA), maintained in lab, and exposed or not to UV rays (*λ* = 254 nm) for 1 min. Results show us that DNA damages were 19.05 ± 9.59% for negative controls and 42.35 ± 9.43 for positive controls. An ANOVA test showed that DNA damages of negative controls were significantly significant compared to positive controls (*p* < 0.01).

The optimal concentration of hemocytes for the comet assay was between 600,000/mL and 1,000,000 cells/mL to avoid overlapping comets and ensure the fast scoring of results. First, 100 µL of cell suspension was mixed with an equal volume of 1% (*w/v*) Low Melting Point (LMP, type VII) agarose in PBS Ca^2+^ and Mg^2+^ free (10 mM, pH 7.4, 37 °C). Two 30 µL-drops (gel replicates) of the previous mixture were deposited on a superfrosted microscopic slide, previously precoated with 0.8% (*w/v*) Normal Melting Point (NMP, Type I) agarose, which were then covered with a coverslip. Slides were cooled for 10 min until the agarose layer harden. After agarose solidification, the coverslip was removed and microscopic slides were placed in a Coplin jar filled with a cold lysing solution (2.5 M NaCl, 100 mM Na_2_EDTA, 10 mM Tris, pH 10, with 1% Triton X100 and 10% DMSO added immediately before use) for at least 1 h at 4 °C in the dark. After cell lysis, slides were rinsed with chilled buffer and then were gently placed in a horizontal electrophoresis chamber (24 cm × 27 cm × 7 cm; W:L:H) filled with a freshly prepared chilled buffer (i.e., 300 mM NaOH, 1 mM Na_2_EDTA, pH > 13). Electrophoresis was performed at 0.83 V/cm for 24 min to unwind DNA. After electrophoresis, slides were washed for 20 min in a neutralization buffer (0.4 M Tris-HCl, pH 7.5) before being dehydrated in absolute ethanol (5 min). Each slide was then stained by adding a 30 µL-drop on each gel with a 1X SYBR Green I dye solution according to manufacturer’s recommendation and then observed under an epifluorescence microscope (Laborlux S, Leica^®^, Wetzlar, Germany) equipped with the appropriate dichroic filter. For each slide, a minimum of 50–100 comets was blindly and randomly scored on the central part of the gel. The Tail intensity parameter given by the Comet assay IV software (Perceptive Instruments^®^, Bury Saint Edmunds, UK), corresponding to the percentage of DNA in the tail of the comet (% tDNA), was chosen for expressing DNA damage. According to the non-normal distribution of comet assay data (% tailDNA), we have considered the median value for each replicate or gel for a mussel. Finally, the mean of the two medians for a mussel (2 replicates or gels by mussel) has been considered in the statistical analysis.

### 5.5. Statistics 

Statistical analysis was performed with Statistica (Version 8.0.360.0, Statsoft, Tulsa, OK, USA, 2007). The normality has been studied with a Shapiro-Wilk test, and the homogeneity of variances was studied with a Levene test. To compare controls and exposed individuals of a same sampling time, a Kruskall-Wallis test has been done. Then, when the p value obtained was significant (*p* < 0.01), a Mann-Whitney test was done. To compare multiples independent conditions, a Kruskall-Wallis test was performed. Here, only data with *p* < 0.01 were considered as significant.

## Supplementary Materials

The following are available online at http://www.mdpi.com/2072-6651/10/3/106/s1, Figure S1: UHPLC-MS/MS Chromatogram of analytical standards, Figure S2: UHPLC-MS/MS Chromatogram of hemolymph samples exposed to 7.5 µg of dissolved BMAA/mussel/3 days.
