# Supplementary material for: Genotoxic and Cytotoxic Effects on the Immune Cells of the Freshwater Bivalve Dreissena polymorpha Exposed to the Environmental Neurotoxin BMAA
Source: Toxins (Basel). 2018 Mar 1;10(3):106. doi: 10.3390/toxins10030106 (PMC5869394; doi:10.3390/toxins10030106)
Supplement: Supplementary file 1 [file toxins-10-00106-s001.pdf]

Supplementary Materials: Genotoxic and Cytotoxic Effects on the Immune Cells of the Freshwater Bivalve Dreissena polymorpha Exposed to the Environmental Neurotoxin BMAA

Alexandra Lepoutre, Nadia Milliote, Marc Bonnard, Mélissa Palos-Ladeiro, Damien Rioult, Isabelle Bonnard, Fanny Bastien, Elisabeth Faassen, Alain Geffard and Emilie Lance

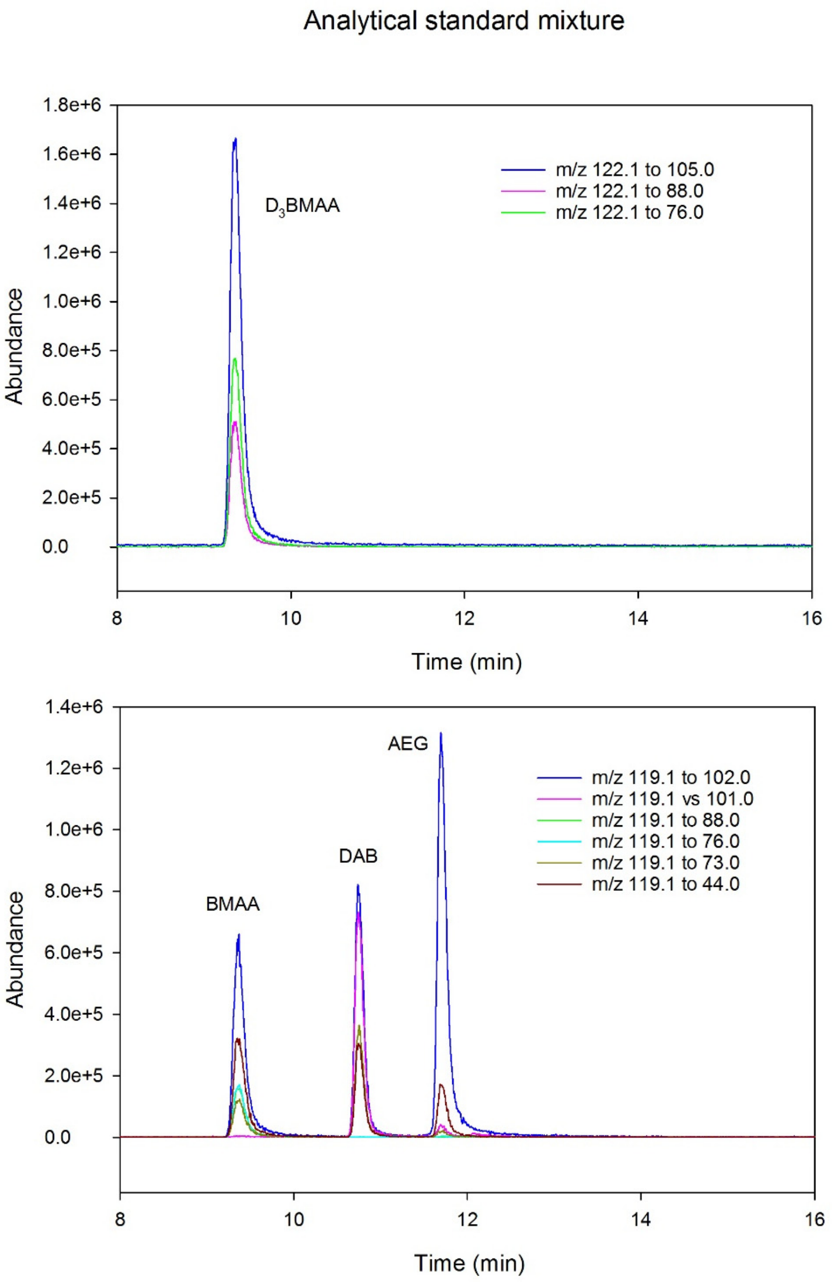

Figure S1. UHPLC-MS/MS Chromatogram of analytical standards.

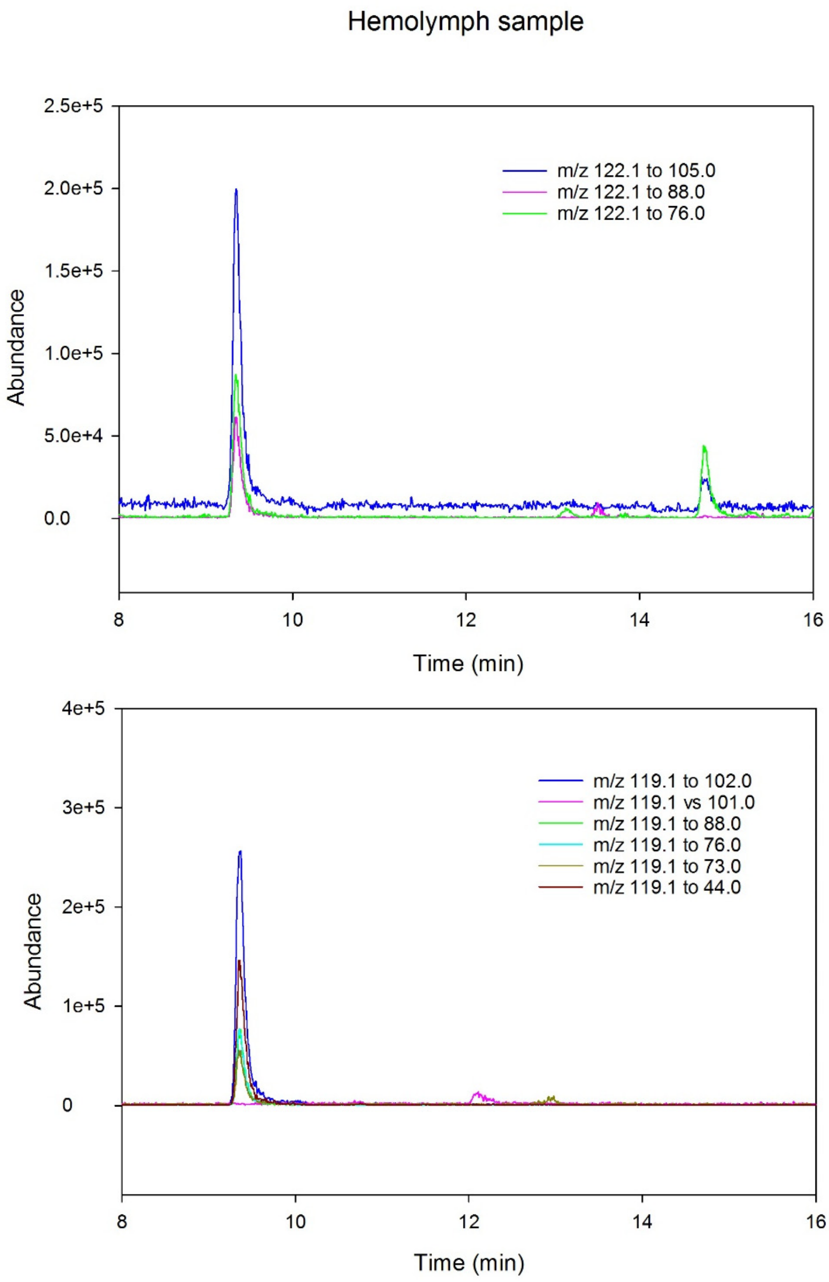

Figure S2. UHPLC-MS/MS Chromatogram of hemolymph samples exposed to 7.5 μg of dissolved BMAA/mussel/3 days.
